# Wertigkeit der CT und des transthorakalen Lungenultraschalls bei PatientInnen mit systemischer Sklerose: Gemeinsame Stellungnahme der ÖRG/ÖGP/ÖGR/ÖGUM

**DOI:** 10.1007/s00393-022-01206-4

**Published:** 2022-05-05

**Authors:** M. Grohs, F. C. Moazedi-Fuerst, H. Flick, K. Hackner, A. Haidmayer, S. Handzhiev, H. Kiener, J. Löffler-Ragg, G. Mathis, G. Mostbeck, O. Schindler, G. Widmann, H. Prosch

**Affiliations:** 1 BVAEB – Rehabilitationszentrum Engelsbad, Weilburgstr. 7–9, 2500 Baden, Österreich; 2 Klinische Abteilung für Rheumatologie und Immunologie, Medizinische Universität Graz, Auenbruggerplatz 15, 8036 Graz, Österreich; 3 Klinische Abteilung für Pneumologie, Medizinische Universität Graz, Auenbruggerplatz 15, 8036 Graz, Österreich; 4 Klinische Abteilung für Pneumologie, Universitätsklinikum Krems, Karl Landsteiner Privatuniversität für Gesundheitswissenschaften, Mitterweg 10, 3500 Krems an der Donau, Österreich; 5 Landeskrankenhaus Südoststeiermark, Dr.-Schwaiger-Str. 1, 8490 Bad Radkersburg, Österreich; 6 Klinische Abteilung für Pneumologie, Universitätsklinikum Krems, Karl Landsteiner Privatuniversität für Gesundheitswissenschaften, Mitterweg 10, 3500 Krems an der Donau, Österreich; 7 Universitätsklinik für Innere Medizin III / Rheumatologie, Medizinische Universität Wien, Währinger Gürtel 18–20, 1090 Wien, Österreich; 8 Universitätsklinik für Innere Medizin II / Pneumologie, Tirol Kliniken GmbH – Medizinische Universität Innsbruck, Anichstr. 35, 6020 Innsbruck, Österreich; 9 Bahnhofstr. 16, 6830 Rankweil, Vorarlberg Österreich; 10 Evangelisches Krankenhaus, Schopenhauerstr. 14, 1180 Wien, Österreich; 11 Abteilung für Innere Medizin und Pneumologie, Standort Enzenbach, LKH Graz II, Gratwein-Strassengel, Österreich; 12 Universitätsklinik für Radiologie, Tirol Kliniken GmbH – Medizinische Universität Innsbruck, Anichstr. 35, 6020 Innsbruck, Österreich; 13 Universitätsklinik für Radiologie und Nuklearmedizin, Medizinische Universität Wien, Währinger Gürtel 18–20, 1090 Wien, Österreich

**Keywords:** Lungenbeteiligung, Transthorakaler Lungenultraschall, Risikofaktoren, Lungenfibrose, Frühdiagnose, Lung involvement, Transthoracic lung ultrasound, Risk factors, Pulmonary fibrosis, Early diagnosis

## Abstract

Die Lungenbeteiligung ist die häufigste Todesursache bei Patienten mit systemischer Sklerose (SSc). Da eine Lungenbeteiligung häufig asymptomatisch ist, wird derzeit empfohlen, bei allen Patienten mit einer neu diagnostizierten SSc eine Thorax-CT durchzuführen. Uneinigkeit herrscht derzeit darüber, wie SSc-Patienten, bei denen zum Diagnosezeitpunkt keine Lungenbeteiligung gefunden wurde, weiterverfolgt werden sollen. Basierend auf einem Konsensus österreichischer Rheumatologen, Pneumologen und Radiologen, wird empfohlen, bei asymptomatischen PatientInnen mit einer negativen CT zum Zeitpunkt der Erstdiagnose jährlich transthorakale Ultraschalluntersuchungen sowie Lungenfunktionsuntersuchungen alle 6 bis 12 Monate durchzuführen. Bei Vorliegen eines positiven Lungenultraschallbefundes wird eine ergänzende CT zur weiterführenden Abklärung empfohlen. Aufgrund der Datenlage werden bei PatientInnen mit einem höheren Risiko, definiert durch entsprechende Risikofaktoren, jährliche CT-Verlaufskontrollen empfohlen.

## Abstract (en)

Lung involvement is the most frequent cause of death in patients with systemic sclerosis (SSc). As lung involvement is frequently asymptomatic, the current recommendation is to carry out thoracic computed tomography (CT) in all patients newly diagnosed with SSc. There is currently disagreement on how patients with SSc for whom no lung involvement was found at the time of diagnosis, should be followed up. Based on a consensus of Austrian rheumatologists, pneumologists and radiologists it is recommended that for asymptomatic patients with a negative CT at the time of initial diagnosis, a transthoracic ultrasound examination should be carried out annually and a lung function examination every 6–12 months. In the presence of a positive lung ultrasound finding a supplementary CT for further clarification is recommended. Based on the data situation, annual CT follow-up controls are recommended for patients with a high risk as defined by appropriate risk factors.

Die systemische Sklerose ist eine seltene Autoimmunerkrankung mit einer Prävalenz zwischen 3 und 24 pro 100.000 Menschen, die neben einer Fibrosierung der Haut auch innere Organe beteiligen kann [19]. In der Lunge führt die systemische Sklerose bei bis zu 80 % der PatientInnen zu einer interstitiellen Lungenerkrankung (ILD) mit Lungenfibrose (SSc-ILD), die bei bis zu 25–30 % der PatientInnen einen progressiven Verlauf einschlägt [19]. Bei bis zu 35 % der PatientInnen ist die Lungenfibrose noch vor der isolierten pulmonalen arteriellen Hypertonie (26 %) die häufigste Todesursache bei Betroffenen mit einer systemischen Sklerose [37]. Nach derzeitigem Wissen ist eine Lungenfibrose irreversibel. Durch eine frühe medikamentöse Therapie kann das Fortschreiten der Erkrankung verlangsamt werden [11]. Aus diesem Grund ist die Frühdiagnose einer ILD bei systemischer Sklerose von besonderer Wichtigkeit.

In einer auf einem Delphi-Prozess basierenden Expertenempfehlung wird aus diesem Grund bei allen PatientInnen mit neu diagnostizierter systemischer Sklerose empfohlen, als Screening eine Computertomographie (CT) des Thorax [17] durchzuführen. Bei PatientInnen mit initial negativer CT sollten gemäß derselben Empfehlung weitere CT-Screening-Untersuchungen in Abhängigkeit von individuellem Risiko, klinischer und lungenfunktioneller Dynamik durchgeführt werden [17].

Da die SSc-ILD ein interdisziplinäres Zusammenarbeiten erfordert, haben sich die Österreichische Gesellschaft für Rheumatologie (ÖGR), die Österreichische Gesellschaft für Pneumologie (ÖGP), die Österreichische Röntgengesellschaft (ÖRG) und die Österreichische Gesellschaft für Ultraschall in der Medizin (ÖGUM) zu einem gemeinsamen Statement zur bildgebenden Diagnostik bei SSc-ILD unter besonderer Berücksichtigung des transthorakalen Ultraschalles entschlossen.

## Methodik

Eine ausführliche Literaturrecherche wurde mit den Begriffen „transthoracic ultrasound and systemic sclerosis“, „transthoracic ultrasound and scleroderma“, „transthoracic ultrasound and interstitial lung disease“, „lung sonography and systemic sclerosis“, „lung sonography and scleroderma“ und „lung sonography and interstitial lung disease“ durchgeführt. Es fanden sich 297 Arbeiten, wobei 44 Literaturstellen in diese Arbeit einflossen. „Case reports“, „case series“ und Arbeiten mit weniger als 25 Probanden wurden nicht hinzugezogen. Die Arbeiten wurden von ExpertInnen aus den ILD-Arbeitskreisen der ÖGP, ÖGR, ÖGUM und ÖGR gelesen. In mehreren Sitzungen wurden Fragen bezüglich der Anwendung des transthorakalen Lungenultraschalls bei systemischer Sklerose abgestimmt. Das Manuskript wurde nach Verfassung von den Vorständen der ÖGP, ÖRG, ÖGUM und ÖGR approbiert. Von keinem der Mitglieder der Konsensusgruppe wurde ein potenzieller Interessenkonflikt angegeben. Für die Erstellung des Konsensus wurde auch seitens der Pharmaindustrie keine finanzielle Unterstützung erhalten.

## Bildgebende Verfahren zur Abklärung einer SSc-ILD

### Thoraxröntgen

Aufgrund ihrer geringen Sensitivität sollten konventionelle Röntgenuntersuchungen des Thorax nicht zur Diagnose oder Verlaufskontrolle von PatientInnen mit SSc-ILD eingesetzt werden.

### Computertomographie (CT) der Lunge

**Figure 1:**
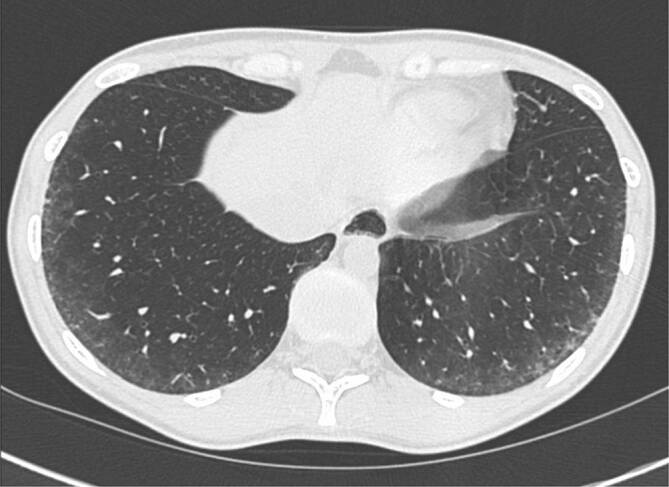

Die Computertomographie ist der Lungenfunktion in der Frühdiagnose einer ILD überlegen [35]. In der CT manifestiert sich die SSc-ILD meist (80 %) unter dem Bild einer nicht-spezifischen interstitiellen Pneumonie (NSIP). Die typischen Veränderungen sind dabei Milchglasverdichtungen neben einer variablen Ausprägung von retikulären Veränderungen in einer basalen und peripheren Verteilung mit häufiger subpleuraler Aussparung (Abb. 1).

**Figure 2:**
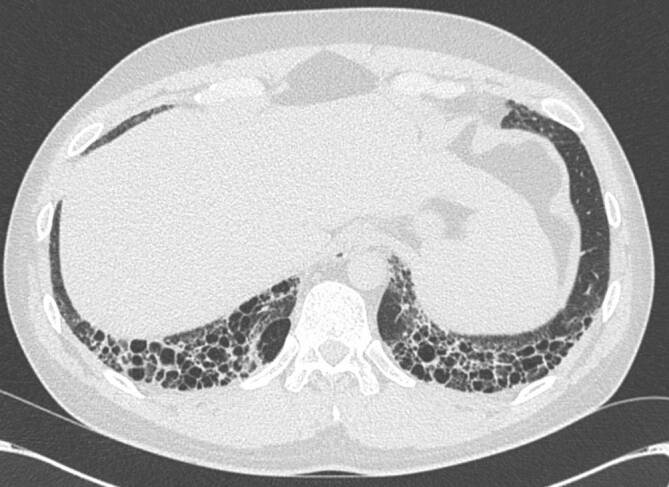

Als Folge der fibrosierenden Veränderungen bei der NSIP finden sich auch ein Volumenverlust der Lunge, eine irreversible Erweiterung von Bronchien (Bronchiektasien) und bei manchen PatientInnen auch ein gering ausgeprägtes Honigwabenmuster (Honeycombing, [8, 10]). Als Honeycombing beschreibt man eine Anhäufung (Cluster) von zahlreichen aneinander grenzenden Zysten ähnlicher Größe, die sich die Wände teilen ([42]; Abb. 2).

Deutlich seltener (10 %) findet sich bei PatientInnen mit SSc-ILD das Muster „usual interstitial pneumonia“ (UIP), welches durch retikuläre Verdichtungen, Traktionsbronchiektasien und Honeycombing in basaler und subpleuraler Verteilung gekennzeichnet ist [8, 10]. Bei weiteren 10 % der PatientInnen finden sich Zeichen einer fibrosierenden Lungenerkrankung, die keinem definierten Muster zugeordnet werden können.

In den letzten Jahren sind auch spezifische Verteilungsmuster bei SSc-ILD beschrieben worden, die insbesondere die anterioren Oberlappen und die „Ecken“ der Lungenlappen als prädominante Bereiche einer Mitbeteiligung identifiziert haben [40].

Das „4-Corner-Sign“ (FCS) in der Computertomographie mit Inflammation/Fibrose im Bereich der bilateralen anterolateralen Oberlappen und posterosuperioren Unterlappen ist außerdem ein spezifisches Zeichen, um eine SSc-ILD von einer *i*diopathischen *p*ulmonalen *F*ibrose abzugrenzen [40].

Neben der Beschreibung des Musters ist auch die Abschätzung des Ausmaßes der Veränderungen von wesentlicher Bedeutung, da dies für die Prognose bestimmend ist. PatientInnen mit einem Ausmaß der Lungenveränderungen von weniger als 20 % des Lungenvolumens haben dabei eine wesentlich bessere Prognose als solche mit mehr als 20 % [13].

Ein weiterer wichtiger Befund in der Thorax-CT ist eine Erweiterung des Truncus pulmonalis, welche auf eine pulmonale Hypertonie hinweist. Die CT kann jedoch nur den Verdacht auf eine PH stellen, weswegen zu einem PH-Screening unter anderem auch eine jährliche Echokardiographie empfohlen wird [3]. Die Abklärung einer pulmonalen Hypertonie ist von Wichtigkeit, da neben der ILD die PH einen bedeutenden Einfluss auf die Mortalität hat [12]. Die PH wird aber in dieser Arbeit nicht weiter behandelt.

CT-Untersuchungen der Lunge bei PatientInnen mit neu diagnostizierter systemischer Sklerose sollten als native Dünnschicht-Volumen-CT (geringste mögliche Kollimation, rekonstruierte Schichtdicke ≤ 1,0 mm) in Rückenlage in tiefer Inspiration mit reduzierter Dosis und hohem Pitch durchgeführt werden (Effektivdosis zwischen 1 und 3 mSv). Zur Vermeidung von diagnostischen Unklarheiten aufgrund von dorsalen Hypostasearealen sollten die PatientInnen im Zweifelsfall zusätzlich in Bauchlage mit kontinuierlichen Schichten oder geringen Untersuchungsvolumina in kraniokaudaler Richtung der Karina untersucht werden. Längere Liegezeiten auf dem Rücken sollten daher nach Möglichkeit vermieden werden. Ergänzende Schichten (kleinste Volumina in Exspiration) sind bei Verdacht auf Obstruktion oder bei unklarem Mosaikmuster in der Inspirations-CT sinnvoll. Iterative Rekonstruktionen der Bilder können verwendet werden [29]. Zur besseren Vergleichbarkeit sollte das Untersuchungsprotokoll der Verlaufsuntersuchungen analog zur Erstuntersuchung erfolgen.

### Magnetresonanztomographie (MRT) und Positronenemissionstomographie (PET)

Obwohl einzelne Studien zur Vorhersage des Krankheitsverlaufes der SSc-ILD mittels MRT oder PET vielversprechende Resultate zeigen konnten [25, 26, 28, 33, 38], empfehlen wir zu diesem Zeitpunkt den Einsatz dieser Modalitäten außerhalb von Studien nicht.

### Transthorakaler Ultraschall

Der transthorakale Lungenultraschall (LUS) ist eine Methode zum Nachweis von Pleura und Parenchymveränderungen und ist ein in der klinischen Routine etabliertes Verfahren zur Diagnose von Lungenödem, Pneumothoraces, Pleuraergüssen u. Ä. [20].

In den letzten Jahren ist der LUS auch vermehrt Thema von Publikationen, die sich mit der Früherkennung, der Diagnose und der Verlaufsbeurteilung einer Lungenbeteiligung bei systemischer Sklerose auseinandersetzen.

Der Ultraschallnachweis einer Lungenbeteiligung bei systemischer Sklerose beruht auf dem Nachweis von Reverberationsartefakten (Nachhallartefakte), welche sich als laserartige vertikale hyperechogene Linien von der Pleura bis zum Bildschirmrand erstrecken und sich synchron mit der Respiration bewegen [39].

**Figure 3:**
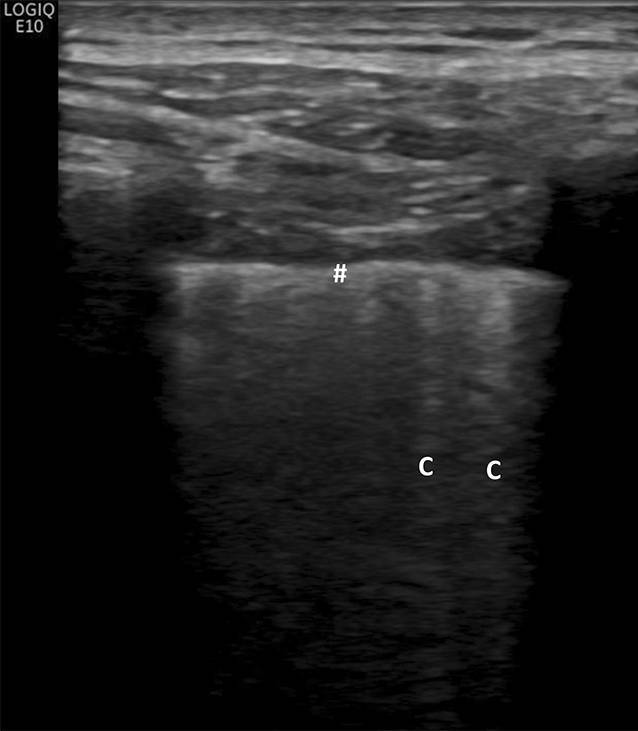

Diese Reverberationsartefakte werden sichtbar, wenn der Luftgehalt im Lungenparenchym teilweise reduziert ist bzw. das Volumen des interstitiellen Raums ausgeweitet ist, wie es im Rahmen des pulmonalen Ödems oder bei interstitiellen Lungenerkrankungen der Fall ist [30–32]. Reverberationsartefakte werden in der Literatur als B‑Linien oder auch als Kometenschweifartefakte beschrieben und sind für sich genommen nicht spezifisch für bestimmte Erkrankungen (Abb. 3). Sie können aber im klinischen Kontext zur Frühdiagnose von Lungenveränderungen bei systemischer Sklerose und anderen interstitiellen Lungenerkrankungen herangezogen werden. In einer Konsensusarbeit der World Federation for Ultrasound in Medicine and Biology (WFUMB) wird empfohlen, Reverberationsartefakte, welche von einer glatten Pleura ausgehen (wie beim Lungenödem), als B‑Linien zu beschreiben, und Reverberationsartefakte, welche von einer verdickten oder unregelmäßigen Pleura ausgehen (wie bei interstitiellen Lungenerkrankungen), als Kometenschweifartefakte („comet tail artefacts“) zu beschreiben [21].

Die diagnostische Wertigkeit des transthorakalen Ultraschalls für SSc-ILD wurde in einer Reihe von wissenschaftlichen Arbeiten untersucht (Tab. 1 und 2) und gegen den Goldstandard CT verglichen. Hierbei wurden die CT-Untersuchungen mit dem Warrick-Score ausgewertet und dem transthorakalen LUS gegenübergestellt. Der Warrick-Score ist eine semiquantitative Scoring-Methode, um das Ausmaß und die Schwere der SSc-ILD in der Computertomographie zu beschreiben [41].

**Table 1:** 

Referenz	Patientenkollektiv	Krankheitsbilder	Goldstandard	Schallkopf	Interkostale Messungen	Sensitivität (B-Lines)	Spezifität (B-Lines)	PPW	NPW
Delle Sedie und Doveri (2010) [9]	25	SSc	HRCT	2,5–3,5 MHz cardiac	62	85 %	70 %	–	–
				6–12 MHz linear		85 %	60 %		
Barskova et al. (2013) [2]	58	SSc	HRCT	2,5–3,5 MHz cardiac sector	72	100 %	55 %	100 %	78 %
Aghdashi et al. (2013) [1]	19 SSc, 8 RA, 2 Overlap-Syndrom, Sjögren-Syndrom, Dermatomyositis	SSc, RA, Overlap, Sjögren-Syndrom, Dermatomyositis	HRCT	7–10 MHz linear	10	73,6 %	88,2 %	95,1 %	51,7 %
Mohammadi et al. (2014) [24]	70	SSc	HRCT	7–10 MHz linear	10	73,6 %	88,2 %	95,1 %	51,7 %
Pinal-Fernandez et al. (2015) [27]	21 ASS, 16 SSc	ASS, SSc	HRCT	5 MHz linear	72	79 % (wenn PI > 24 %)	100 % (wenn PI > 24 %)	–	–
Sperandeo et al. (2015) [34]	175	SSc	HRCT	3,5–5 MHz convex	72	94 %	95,2 %	–	–
						74,3–80 % (PL Verdickung)	99 % (PL Verdickung)		
Çakir Edis et al. (2016) [7]	48	SSc	HRCT	5–10 MHz linear	14	100 %	84,2 %	90,6 %	100 %
Tardella et al. (2017) [36]	40	SSc	HRCT	4–13 MHz linear	14	96,3 %	92,3 %	–	–
Hassan et al. (2019) [16]	67	SSc	HRCT	3,5 MHz convex	72	100 %	34,21 %	53,7 %	100 %

*ASS* Antisynthetasesyndrom, *PI* Pleurairregularitäten, *PL* Pleuradicke

**Table 2:** 

Referenz	Scoring
Delle Sedie und Doveri (2010) [9]	Scoring nach Picano et al. Score 0: < 5 B‑Linien Score 1: 5–15 B‑Linien Score 2: 15–30 B‑Linien Score 3: > 30 B‑Linien
Barskova et al. (2013) [2]	Summe der B‑Linien > 3 B‑Linien in 2 Arealen > 5 B‑Linien insgesamt Kompletter „White screen“ > 10 B‑Linien
Aghdashi et al. (2013) [1]	> 5 B‑Linien positiv
Mohammadi et al. (2014) [24]	> 5 B‑Linien positiv
Pinal-Fernandez et al. (2015) [27]	Anzahl an B‑Linien pro anatomischer Region (Gargani et al.)
Sperandeo et al. (2015) [34]	< 3 B‑Linien negativ > 3 B‑Linien positiv
Çakir Edis et al. (2016) [7]	≥ 3 B‑Linien in einer Region oder > 5 B‑Linien in benachbarten Regionen werden als positiv bewertet. Ein kompletter „White screen“ in einer Region wird als 10 B‑Linien gewertet
Tardella et al. (2017) [36]	Wenige B‑Linien werden einzeln gezählt. Sind diese jedoch konfluierend, wird nach der semiquantitativen Auswertung nach Gargani und Volpicelli ausgewertet, das bedeutet, der Prozentsatz an vorhandenen B‑Linien wird durch 10 dividiert (z. B. 30 % eines „White screen“ korrespondiert zu 3 B‑Linien, 40 % zu 4 B‑Linien und so weiter)
Hassan et al. (2019) [16]	B‑Linien werden gezählt und ausgewertet nach dem Score von Picano et al.

Diese Studien konnten zeigen, dass der LUS bei Patienten mit systemischer Sklerose eine hohe Spezifität und Sensitivität im Nachweis von Lungenveränderungen aufweist und sich somit zur Früherkennung dieser Veränderungen eignet.

Wesentliche Ultraschallmerkmale einer Lungenbeteiligung im Rahmen einer systemischen Sklerose sind Reverberationsartefakte, die von einer unregelmäßigen/fragmentierten Pleuralinie ausgehen und somit die Kriterien von Kometenschweifartefakten erfüllen. Es hat sich gezeigt, dass das Vorhandensein von 10 pro Interkostalraum oder mehr Kometenschweifartefakten als Cut-off-Point mit dem höchsten positiven Vorhersagewert für eine signifikante SSc-ILD entsprechend einem Warrick-Score ≥ 7 angesehen werden kann; die Sensitivität und Spezifität betragen 96,3 % bzw. 92,3 % [36].

**Figure 4:**
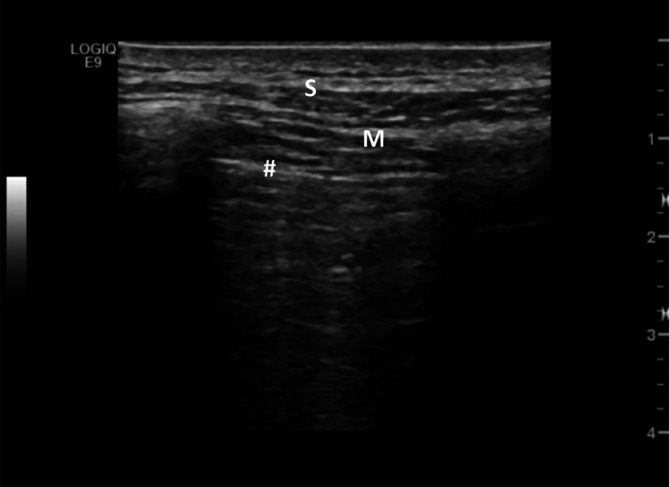

Neben den Kometenschweifartefakten wird auch eine Verbreiterung der Pleuralinie auf ≥ 3 mm in jeglicher Region als pathologisch gewertet [23]. Als Pleuralinie wird die Grenzzone zur belüfteten Lunge beschrieben, bei der es zu einer Totalreflexion der Ultraschallwellen kommt. Obwohl die Pleura selbst aufgrund ihrer geringen Breite nicht durch den Ultraschall dargestellt werden kann, wird die Pleuralinie von vielen Autoren etwas vereinfacht mit der Pleura gleichgesetzt (Abb. 4).

Die im Rahmen einer systemischen Sklerose beobachtete unregelmäßige Verbreiterung der Pleuralinie kann durch die Verbreiterung des pulmonalen Interstitiums im Rahmen der Lungenerkrankung erklärt werden. Eine verbreiterte Pleuralinie findet sich aber nicht nur im Rahmen einer ILD, sondern auch bei Pneumonien sowie manchmal auch bei Pleuritis, Lungenemphysem, Lymphangitis, neoplastischen oder zystischen Lungenerkrankungen.

## Untersuchungsablauf

Beim Lungenultraschall kommen sowohl niedrigfrequente konvexe (3,5–5 MHz) als auch hochfrequente lineare Schallköpfe (8–11 MHz) zur Anwendung. Reverberationsartefakte werden vorzugsweise mit dem konvexen Schallkopf bis zu einer Tiefe von ca. 10 cm dargestellt, während Pleuraveränderungen detaillierter mit dem linearen Schallkopf visualisiert werden können. Im Rahmen einer pulmonalen Fibrose sind Pleurairregularitäten oft die frühesten Veränderungen, aber auch Fragmentation, Verdickung und Unschärfe können zur Darstellung gelangen, die histologisch möglicherweise einer Kombination aus pleuralen und parenchymalen Veränderungen entsprechen. Diese Parameter scheinen eine höhere diagnostische Sensitivität für eine SSc-ILD zu haben und weisen im Vergleich zu Kometenschweifartefakten einen höheren negativen prädiktiven Wert auf [5, 6, 22, 27].

**Figure 5:**
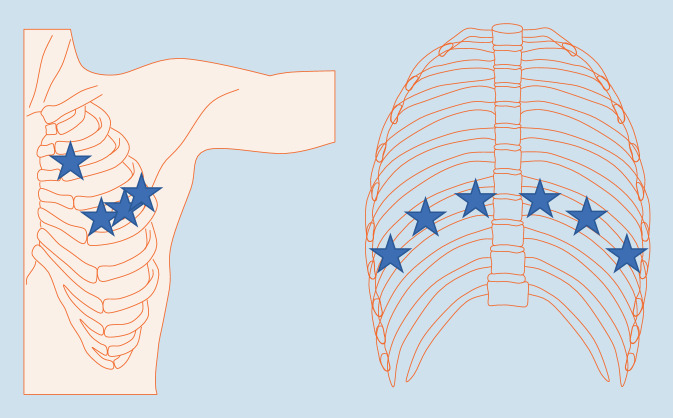

Gutierrez et al. [15] haben in einer Studie die umfassende („comprehensive“) und einfache („simplified“) Lungenultraschalluntersuchung (LUS) verglichen. Beim „comprehensive assessment“ werden 50 LIS („lung intercostal spaces“) und beim „simplified assessment“ 14 LIS untersucht. In der vereinfachten Form werden von anterior der 2. ICR parasternal, der 4. ICR medioklavikulär, der 4. ICR in der vorderen und mittleren Axillarlinie sowie von posterior der 8. ICR im Bereich der paravertebralen, subskapulären und hinteren Axillarlinie untersucht (Abb. 5). Beide Protokolle zeigen eine positive Korrelation der Kometenschweifartefakte mit dem HRCT-Warrick Score. Weiter zeigt sich eine hoch signifikante Korrelation zwischen dem „comprehensive“ vs. „simplified assessment“ („Spearman’s rank test“ *p* = < 0,001).

Die umfassende LUS-Untersuchung dauert im Mittel 23,3 min vs. 8,6 min bei der einfachen LUS-Untersuchung [15].

## Diskussion

Aufgrund ihrer schlechten Prognose und neuen therapeutischen Möglichkeiten ist die Früherkennung einer Lungenbeteiligung im Rahmen einer systemischen Sklerose von besonderer Wichtigkeit. Die frühe Diagnose der Lungenbeteiligung sowie die engmaschige Verlaufskontrolle von PatientInnen mit einer SSc-ILD alle 3 bis 6 Monate je nach Schwere der Erkrankung ermöglichen eine Therapiesteuerung mit ggf. erforderlicher Therapieanpassung.

Bis dato sind bestimmte Risikofaktoren definiert, die eine SSc-ILD begünstigen würden, jedoch liegen keine verlässlichen Parameter vor, um den zeitlichen und klinischen Verlauf (fulminant, chronisch progredient, nicht progredient) der ILD bei SSc voraussagen zu können. Obwohl Lungenfunktionstests sowohl in der Früherkennung als auch im Verlauf von diffusen parenchymatösen Lungenerkrankungen bei Kollagenosen einen wichtigen Stellenwert haben, sind sie v. a. zu Beginn nicht ausreichend sensitiv und spiegeln oftmals nicht den Fibrosegrad wider [4]. Der Stellenwert der Lungenfunktion liegt daher v. a. in der Verlaufsbeurteilung. Die wichtigsten funktionellen Parameter zur Diagnose und Verlaufsbeurteilung einer Lungenmitbeteiligung sind dabei die forcierte Vitalkapazität (FVC) und die Diffusionskapazität (DLCO). Diese Werte können bei den einzelnen Messungen variieren, sodass der Verlaufstrend wichtiger als die Einzelmessungen für die Beurteilung einer Lungenbeteiligung bei der SSc ist. Da die normale FVC (> 80 % des Sollwertes bzw. > „lower limit oft normal“ [LLN]) eine Falsch-negativ-Rate von bis zu 62,5 % aufweist, wäre dieser Messwert, allein genommen, nicht zur Früherkennung einer Lungenbeteiligung geeignet [35]. Auch durch eine Kombination von FVC, ∆FVC, TC, DLCO und FEV_1_/FVC kann die Falsch-negativ-Rate lediglich auf 27 % gesenkt werden [35]. Während Lungenfunktionsparameter für die Frühdetektion einer SSc-ILD somit eine niedrige Sensitivität aufweisen, sind sie als Prognoseparameter im Verlauf der Erkrankung gut geeignet [14].

Aus diesem Grund wird in einer rezenten Expertenempfehlung neben einer Lungenfunktion bei allen PatientInnen mit einer neu diagnostizierten systemischen Sklerose eine CT des Thorax zum Ausschluss oder zur Diagnose einer Lungenmitbeteiligung empfohlen [17]. Dieselbe Expertengruppe konnte sich hinsichtlich der Frage, ob bei initial negativer CT weiterführende Früherkennungsuntersuchungen mittels transthorakalen Lungenultraschalls durchgeführt werden sollten, nicht einigen.

Obwohl sich bereits eine Reihe von Arbeiten mit dem LUS bei systemischer Sklerose auseinandergesetzt hat, muss hier kritisch angemerkt werden, dass die Rolle des LUS als Früherkennungstool bei asymptomatischen PatientInnen bisher noch nicht ausreichend untersucht wurde und prospektive Studien hier noch ausstehen. Die publizierten Daten zur Detektion von pulmonalen Veränderungen bei systemischer Sklerose und anderen diffusen parenchymatösen Lungenerkrankungen legen aber nahe, dass der LUS als nichtinvasive Methode geeignet wäre, frühe Veränderungen bei asymptomatischen PatientInnen zu erfassen.

Die österreichische Expertengruppe empfiehlt daher, basierend auf den publizierten Studien zu diesem Thema und langjähriger Erfahrung der Experten, jährlich transthorakale Ultraschalluntersuchungen bei asymptomatischen PatientInnen mit einer negativen CT zum Zeitpunkt der Erstdiagnose der systemischen Sklerose durchzuführen. Der negative Vorhersagewert des LUS in dieser Fragestellung wurde noch nicht ausreichend studiert, daher sollten – in Anlehnung an die Empfehlungen der europäischen Expertengruppe [18] – insbesondere bei PatientInnen mit einem höheren Risiko für eine SSc-ILD CT-Verlaufskontrollen durchgeführt werden. Das höhere Risiko wird durch das Vorhandensein von Scl-70-AK, die diffuse Verlaufsform der Sklerodermie, respiratorische Symptome und das männliche Geschlecht bestimmt. CT-Kontrollen sollten vor diesem Hintergrund, aber auch durch das Auftreten von Symptomen bzw. bei einer entsprechenden Dynamik der Lungenfunktionsparameter erfolgen. Die Mehrheit der SklerodermiepatientInnen würde diese Risikofaktoren für diese CT-Verlaufskontrollen nicht erfüllen. Daher einigte sich die österreichische Expertengruppe auf jährliche LUS-Untersuchungen bei PatientInnen ohne Dynamik in den 6‑ bis 12-monatigen Lungenfunktionsuntersuchungen und ohne klinische Symptomatik. In Anlehnung an das Consensus Statement von Hoffmann-Vold et al. sollen bei durch oben genannte Faktoren definierten RisikopatientInnen CT-Verlaufskontrollen erfolgen [18]. Die Expertengruppe empfiehlt hier bei Patienten mit einem hohen Risiko jährliche CT-Verlaufskontrollen.

Bei der Lungenultraschalluntersuchung sollten zumindest die posterior basalen (8. ICR) sowie ventrolateralen Anlotungen (2. + 4. ICR) entsprechend dem simplifizierten Assessment erfolgen, da v. a. die Basen der Lungen und die Oberlappen Veränderungen in der CT zeigen.

Als positiv im Sinne unphysiologischer Zeichen gelten im Lungenultraschall im Besonderen Kometenschweifartefakte und/oder Pleuraunregelmäßigkeiten mit einer Verbreiterung von > 3 mm. Zur Darstellung des Lungenparenchyms dient der konvexe Schallkopf, zur Darstellung der Pleura der lineare Schallkopf. PatientInnen mit einem positiven Ultraschallbefund sollten zur weiteren Abklärung und zur Prognoseabschätzung durch eine HRCT abgeklärt werden.

Die Lungenfunktionsparameter sowie die klinische Visite werden in Anlehnung an die Empfehlungen der Expertengruppe je nach Krankheitsaktivität alle 3 bis 6 Monate bei progredienter Erkrankung bzw. alle 6 bis 12 Monate bei einem stabilen Krankheitsverlauf empfohlen [18].

**Figure 6:**
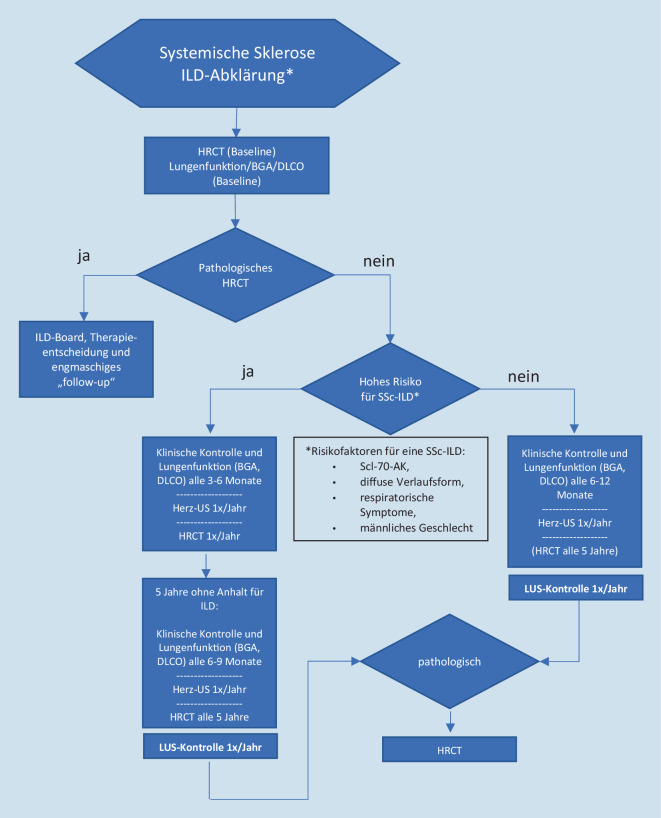

Verlaufsuntersuchungen bei PatientInnen mit bereits diagnostizierter SSc-ILD sollten mittels CT durchgeführt werden, da die Datenlage zum Ultraschall in dieser Fragestellung der Surveillance oder Progredienz noch dürftig ist. Die Expertengruppe einigte sich, dass eine HRCT zumindest alle 5 Jahre auch bei asymptomatischen PatientInnen empfohlen werden sollte (Abb. 6).

## Schlussfolgerung

Die Früherkennung einer ILD bei der systemischen Sklerose ist aufgrund ihrer schlechten Prognose von besonderer Bedeutung. Im Rahmen der Erstdiagnose einer systemischen Sklerose wird bei allen PatientInnen eine CT des Thorax zur Erfassung einer Lungenbeteiligung empfohlen.

Die österreichische Expertengruppe empfiehlt, jährlich transthorakale Ultraschalluntersuchungen bei asymptomatischen PatientInnen mit einer negativen CT zum Zeitpunkt der Erstdiagnose einer systemischen Sklerose, ohne klinische Symptome und ohne Dynamik in den 6‑ bis 12-monatigen Lungenfunktionsparametern durchzuführen. Bei Vorliegen eines positiven Lungenultraschallbefundes wird eine erneute HRCT als nicht-invasiver Goldstandard der Diagnose einer ILD zur weiterführenden Abklärung empfohlen. Aufgrund der Datenlage werden generell bei PatientInnen mit einem höheren Risiko, definiert durch entsprechende Risikofaktoren, für eine SSc-ILD jährliche CT-Verlaufskontrollen empfohlen.
